# Protective Effects and Metabolic Regulatory Mechanisms of Shenyan Fangshuai Recipe on Chronic Kidney Disease in Rats

**DOI:** 10.1155/2020/5603243

**Published:** 2020-08-25

**Authors:** Xinqi Deng, Nan Jiang, Li Guo, Chunguo Wang, Jiaoyan Li, Xiaoci Liu, Bixiu Zhu, Ruiyi Cai, Yanming Chen, Teng Wang, Lanying Liu

**Affiliations:** ^1^School of Life Science, Beijing University of Chinese Medicine, Beijing 100029, China; ^2^Department of Nephropathy, Wuhan Hospital of Traditional Chinese and Western Medicine, Wuhan 430000, China; ^3^Department of Nephropathy, Third Affiliated Hospital of Beijing University of Chinese Medicine, Beijing 100029, China; ^4^Beijing Research Institute of Chinese Medicine, Beijing University of Chinese Medicine, Beijing 100029, China; ^5^Department of Internal Medicine, Shahe Hospital, Beijing 102206, China

## Abstract

**Background:**

Chronic kidney disease (CKD) is one of the major causes of renal damage. Shenyan Fangshuai Recipe (SFR), a modified prescription of traditional medicine in China, showed potent effects in alleviating edema, proteinuria, and hematuria of CKD in clinical practices. In this study, we aimed to investigate scientific evidence-based efficacy as well as metabolic regulations of SFR in CKD treatment.

**Materials and Methods:**

The effect of SFR on CKD was observed in a rat model which is established with oral administration of adenine-ethambutol mixture for 21 days. Further, metabolites in serum were detected and identified with ultra-performance liquid chromatography-high resolution mass spectrometry (UPLC-HRMS). Metabolomics study was performed using Ingenuity Pathway Analysis (IPA) software.

**Results:**

With H&E staining and Masson's trichrome, the results showed that chronic kidney damage is significantly rescued with SFR treatment and recovered to an approximately normal condition. Along with 44 differential metabolites discovered, the regulation of SFR on CKD was enriched in glycine biosynthesis I, mitochondrial L-carnitine shuttle pathway, phosphatidylethanolamine biosynthesis III, sphingosine-1-phosphate signaling, L-serine degradation, folate transformations I, noradrenaline and adrenaline degradation, salvage pathways of pyrimidine ribonucleotides, cysteine biosynthesis III (Mammalia), glycine betaine degradation, and cysteine biosynthesis/homocysteine degradation. Further, TGF*β*-1 and MMP-9 were observed playing roles in this regulatory process by performing immunohistochemical staining.

**Conclusion:**

SFR exerts potent effects of alleviating glomerular sclerosis and interstitial fibrosis in the kidney, mainly via integrated regulations on metabolism and production of homocysteine, L-carnitine, and epinephrine, as well as the expression of TGF*β*-1. This study provides evidence for SFR's protective effects on CKD and reveals the metabolic mechanism behind these benefits for the first time.

## 1. Introduction

Chronic kidney disease (CKD), a set of disorders destroying the structure and function of the kidney [1], is becoming a public health problem worldwide along with increasing incidence and prevalence, poor outcomes, and high cost [2]. CKD could lead to not only kidney failure but also cardiovascular disease [3]. In the early stage of CKD, patients are easily misdiagnosed due to the lack of clinical features; as a result, they would have a high tendency of various vascular complications and uremia if not treated in time. Clinically, serum creatinine (SCr) and serum urea nitrogen (BUN) are commonly used to evaluate renal function, but the indicators are nonspecific. CKD can be caused or aggravated by various risk factors. Hyperuricemia but not serum uric acid levels had been observed to be a risk factor for all-cause mortality and CVD mortality in CKD [4, 5]. The pathological response to hyperuricemia in the kidney had also been well elucidated, including tubular obstruction, tubular cell damage, and subsequent interstitial fibrosis [6, 7]. Recently, multiple mechanisms leading to CKD have been introduced, including but not limited to endothelial dysfunction, oxidative stress, vascular smooth muscle cell proliferation, and inflammatory chemokine factors' expression and activation [8–10].

The pathomechanism of CKD is complex, which makes it difficult to achieve desired curative effects by only applying single-target drugs. Currently, urate-lowering drugs contain 4 main categories including xanthine oxidoreductase (XOR) inhibitors, uricosurics, URAT1 inhibitors, and recombinant uricases. Among them, allopurinol, an inhibitor of XOR, is the drug of choice [11]. However, it may induce clinically unfavorable side effects [12], and the symptoms of CKD are difficult to relieve. Seeking references for effective solutions for diseases from Traditional Chinese Medicine (TCM) has become an attractive point of providing a new perspective for the development of drugs. It has been observed in a large number of clinical studies that Chinese medicine can effectively alleviate hyperuricemia, resist kidney fibrosis, and therefore delay the progression of CKD [13]. However, the research on the mechanism of TCM in the treatment of CKD is extremely limited. SFR has been used for decades and is known for its efficiency in treating and relieving the clinical symptoms including edema, albuminuria, and hematuria of CKD patients. Thus, we hypothesized that the SFR functions on CKD by arresting hypertension, renal interstitial fibrosis, and renal anemia. However, the active mechanism is unknown.

Metabolomics is one of the disciplines of system biology representing the analysis of known and unknown metabolic pathways and has grown tremendously over the past 20 years. It can analyze multiple metabolites of the body in the physiological and pathological states, provides reliable data for exploring metabolic pathways, and has been widely applied in the studies of cardiovascular diseases, endocrine system diseases, and digestive system diseases [14]. However, the serum metabolic profiling of CKD remains poorly understood and pharmacometabolomic assessment of TCM in CKD is hitherto unknown. In this study, we aimed to provide more metabolic evidence for the explanation of SFR's protective effect in an experimental way. The metabolic character of CKD was profiled with UPLC-HRMS and IPA was applied for metabolomic analysis and exploration of SFR's regulatory mechanism in delaying the development of CKD. Effects of uric acid on kidney and mechanisms of hyperuricemia inducing CKD were well illustrated [15]. The CKD rat model employed in this study was founded on hyperuricemia which is induced by the intake of adenine and ethambutol [16, 17]. Serum samples were collected for UPLC-HRMS-based metabolomic observation. Data was processed with principal component analysis (PCA) and orthogonal partial least squares discrimination analysis (OPLS-DA). In conclusion, this study provided evidence of the mechanism of SFR's protection against CKD to lay a foundation for clinical treatment programs. It is also a scientific example to highlight the power of the UPLC-HRMS system and IPA software for sophisticated biological data processing.

## 2. Materials and Methods

### 2.1. Chemicals and Instruments

Adenine (No. HV435-10G) was obtained from Glenview Co., Ltd. (Florida, America), and ethambutol (No. H33021602) was purchased from Hangzhou Minsheng Pharmaceutical Co., Ltd. (Hangzhou, China). The immunohistochemical kits were from Boster Biological Technology co., Ltd. (Wuhan, China). P800 automatic biochemical analyzer was from Roche Diagnostics Ltd. (Penzberg, Germany). Double distilled water (18.2 MΩ·cm) was from the Milli-Q system (Millipore, Bedford, MA). UltiMate 3000 Ultra Performance Liquid Chromatograph and Cortecshilic column (1.6 *μ*m 2.1 × 100 mm) were from Waters Co., Ltd., Massachusetts, USA. TGL 20 M high speed refrigerated centrifuge was from Thermo Fisher Scientific Co., Ltd. (Rockford, USA).

### 2.2. Preparation of SFR Sample

SFR comprises 15 herbs: 9.3% *Astragalus membranaceus* (Fisch.) Bge, 3.1% *Angelica sinensis* (Oliv.) Diels, 9.3% *Spatholobus suberectus* Dunn, 9.3% *Trachelospermum jasminoides* (Lindl.) Lem, 18.6% *Smilax glabra Roxb*, 6.2% *Lilium brownie* F. E. Brown var. *viridulum* Baker, 1.9% *Whitmania pigra* Whitman, 9.3% *Curcuma phaeocaulis* Val, 9.3% *Salvia miltiorrhiza* Bge, 3.1% *Citrus reticulata* Blanco, 1.9% *Citrus reticulata* Blanco, 1.9% *Commiphora myrrha* Engl, 4.6% *Rehmannia glutinosa* Libosch, 3.1% *sargassum fusiforme* (Harv.) Setch, and 9.3% *Ostrea gigas* Thunberg. The herbs were provided by Beijing Kangrentang Pharmaceutical (Beijing, China) and identified by the Department of Pharmacognosy, Beijing University of Chinese Medicine (Beijing, China).

Preparation of SFR samples was as follows: 3.4 kg crude herb of SFR was boiled twice for 2 hours each time with 10 times (w/v) of distilled water. The combined extraction was filtered and then concentrated at 80°C under reduced pressure for 6 hours. After vacuum drying, 1.1 kg dry powder was obtained with a yield ratio of 32.4%. The test doses of SFR for animal experiments were calculated as follows. The dose of SFR in raw materials for an adult human was 28.70 g/day. The body weight of adult human was calculated as 70 kg. Based on the extract ratio of 32.4% of dry powder/raw materials of SFR, the dose of dry powder for the adult human was 9.29 g/kg. To calculate the dose for animals, the ratio of equivalent dose based on surface area converted between humans and rats was 6 : 1. Accordingly, the administration dose of SFR was 1.55 g/kg/d.

### 2.3. Animals Grouping and Modeling

30 male Sprague Dawley rats (8 weeks old, weighting 200 g ± 10 g, Vital River Laboratory Animal Co. Ltd, Beijing, China, Animal certificate SCXK number: 2016-0002) were housed in the clean level condition animal housing facilities (certification number SYXK (Jing) 2016-0042) of BUCM, and temperature (23 ± 2°C), humidity (55 ± 5%), and day/night cycle (12 hours/12 hours) were controlled, with free access to standard laboratory diet (Vital River Laboratory Animal Co. Ltd, Beijing, China) and water. They were randomly divided into control group, model group, and SFR group with 10 rats in each group. After adaptive growth for 7 days, the model group and SFR group were conducted referring to the method reported previously [18, 19] and orally given suspension mixture of 100 mg/kg adenine and 250 mg/kg ethambutol hydrochloride body weight/day; the control group was orally given the same volume of distilled water. After modeling for 3 weeks, the SFR group was orally given SFR sample for 4 weeks, while the control group and model group were given the same volume of distilled water.

### 2.4. Biochemical Analysis

During the modeling process, blood samples were taken from the eyelids of each group, and contents of SCr and SUA were detected by applying a P800 automatic biochemical analyzer on the 14th and 21st day. 4 weeks after SFR administration, rats were anesthetized with 1.5% isoflurane in 70% N_2_O/30% O_2_ (flow rate, 1.0 L/min). Blood was taken from abdominal aorta and was collected in tubes containing sodium heparin and then centrifuged at 3500 rpm for 10 min at 4°C. The serum was collected and stored at −80°C before UPLC analysis.

### 2.5. Pathological Histology

After blood collection, the cortex of the left kidney of the rats was taken and fixed with 4% paraformaldehyde/PBS buffer, dehydrated with ethanol, and then embedded in paraffin. The kidneys were sectioned into slices of 4 *μ*m thickness, and hematoxylin & eosin (H&E) and Masson's trichrome staining were performed, respectively, according to the manufacturer's instructions on the renal tissue sections to examine pathological changes under the light microscope. Ten separate fields for each specimen were randomly selected to evaluate renal impairment with averaged values for the histological score.

Semiquantitative scoring of glomerular sclerosis was performed by two independent trained observers with five-grade criterion described previously [20, 21]: 0 = normal; 1 = mild, the involvement of less than 10% of the cortex; 2 = moderate, the involvement of 10 to 25% of the cortex; 3 = severe, the involvement of 25 to 50% of the cortex; 4 = very severe, the involvement of 50–75% of cortex; 5 = extensive damage, the involvement of more than 75% of the cortex.

### 2.6. Immunohistochemical Staining

The slides were dewaxed, rehydrated, and then microwaved (92°C–98°C) for 10–15 min in a sodium citrate buffer (PH 6.0) to retrieve antigen epitopes. Endogenous peroxidase activity was suppressed by 3% H_2_O_2_ and blocked by 10% goat serum. Diluted primary antibodies against matrix metalloproteinase 9 (rabbit Anti-MMP9 antibody, ab38898, Abcam; 1 : 400) and transforming growth factor-beta (Rabbit Anti-TGF Beta 1 Polyclonal antibody, bs-0103R, Bioss; 1 : 100) were added and placed at 4°C overnight. As secondary reagents, biotin-labeled secondary antibodies were used for 20 min at 37°C; afterward, SABC was filled for another 20 min at 37°C followed by staining with DAB reagent until a brown color developed. Slides were counterstained with hematoxylin and differentiated in hydrochloric acid ethanol. Sections were dehydrated and a transparent coverslip was added to enable observation by microscopy. All samples were blindly inspected by two independent pathologists. Positive immunostaining was visualized as brown granules contained in the cytoplasm. MMP-9 was scored by NIH ImageJ software based on the percentage of the positively stained area as previously reported [22]. OD (optical density) of TGF*β*-1 was measured with spectrodensitometer for semiquantitative analysis for the intensity of immunohistochemistry. All images were analyzed by Image-Pro plus 6.0.

### 2.7. Serum Preparation and UPLC-HRMS Analysis

Serum sample preparation was made by protein precipitation with acetonitrile. 400 *μ*L of precipitant [V (acetonitrile): V (formic acid) = 3 : 1] and 200 *μ*L of serum were added to 1.5 mL centrifuge tube; then, the mixture was vortexed for 2 min. After 10 min precipitation, the mixture was centrifuged at 10,000 rpm for 15 min, the supernatant was transferred and dried on a nitrogen blower, the dried supernatant was redissolved in 100 *μ*L mobile phase (A phase 0.1% formic acid aqueous solution), and pooled QC samples were obtained by mixing equal proportions of all the samples.

Metabolomic analysis was performed in triplicate on Thermo Scientific Dionex Utimate 3000 UHPLC plus Focused coupled to an LTQ/Orbitrap MS system equipped with an electrospray ionization source operating. A 2.1 mm × 100 mm CORTECS UPLC HILIC Column was equipped for all analyses. The injection volume was 2 *μ*L, the flow rate was 400 *μ*L/min, and the column temperature was 30°C. Under the positive ion model, the HESI ion source was applied. Ion source temperature was 350°C; ionization source voltage, 4 kV; capillary voltage, 35 V; tube lens voltage, 110 V; sheath gas and auxiliary gas were high purity nitrogen (purity > 99.99%); sheath gas flow rate, 40 arb; auxiliary gas flow velocity, 20 arb. The data acquisition level adopted Fourier transform high resolution full scan (TF, Full scan, Resolution 30000), and HRMS adopted data dependence, CID mode. UPLC gradient conditions: 0–2 min, 3% solvent B; 2–6 min, 3–15% B; 6–14 min, 15% B; 14–17 min, 15–50% B; 17.1–20 min 3% B.

### 2.8. Data Analysis

The mass spectral raw data obtained from UPLC-HRMS was imported into Sieve 2.1 software (Thermo Fisher Scientific Inc., San Jose, CA, USA) for pretreatment, and the acquired data matrices were unitary processed. The preprocessed data were analyzed by Principal Components Analysis (PCA) and Orthogonal Partial Least Squares-Discriminant Analysis (OPLS-DA), by the application of SIMCA-P13.0 software (Umetrics). Then, the identification of differential metabolites was performed by Compound Discoverer 2.0 for spectrogram matching, combined with the Metlin and HMDB databases. The obtained differential components were analyzed through MeV (Multi Experiment Viewer, v4.8, TIGR) for hierarchical cluster analysis and K-mean cluster analysis. All experimental data were expressed as means ± SD, statistical significance was calculated by Student's *t*-test in the SPSS version 22.0 software package, and *p* values lower than 0.05 were considered significant. IPA software was applied for metabolomics analysis of regulatory pathways.

## 3. Results

### 3.1. SFR Exerted Potent Protective Effects on CKD Model Induced by Hyperuricemia

**Figure 1:**
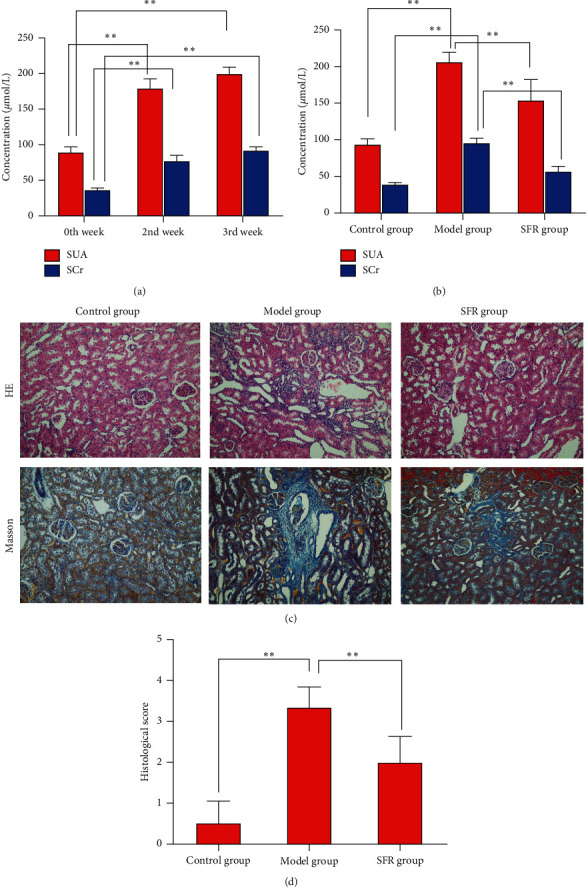
Protective effects of SFR on kidneys of CKD rats. (a) SCr and SUA content variation during the modeling process. (b) SCr and SUA content of rats in control, model, and SFR groups (c) Renal lesions shown with H&E and Masson staining. (d) Score for characteristic histologic signs of renal injury in control, model, and SFR group. Data are presented as mean ± SD, *t*-test, ^*∗∗*^*p* < 0.01.

The content of SCr and SUA in blood was kept increasing during the modeling process (Figure 1(a)), indicating the validity of the CKD model. Under SFR treatment, the content of SCr and SUA in blood was significantly downregulated (Figure 1(b)). With H&E and Masson staining, expanded or atrophied kidney tubules, inflammatory cell infiltration and fibrosis in tubulointerstitium, glomerular sclerosis, and severely dilated capillaries with sinusoid formation were observed in CKD group. Such histopathological damage was dramatically reversed and the renal structure was recovered to a great extent with SFR treatment (Figure 1(c)). Besides, the rescued scores for characteristic histologic signs of renal injury also indicated the protective effects of SFR on CKD (Figure 1(d)).

### 3.2. Statistical Analysis of UPLC-HRMS Data

The signal responses of serum metabolites in ESI+ were collected to analyze the contours of metabolites. The stability of the LC-MS system was checked and optimized by evaluating relative standard deviation (*RSD*) with six duplicate injections of the QC sample. As shown in Table 1, the RSD of the retention times for the precision, and stability were 0.02∼0.34% and 0.10∼0.27%; the RSD of the peak intensity for the precision, and stability were 1.28∼3.68% and 1.11∼1.85%. The results of the precision and stability demonstrated that the proposed method is a robust method and the analysis in the study is satisfied.

**Figure 2:**
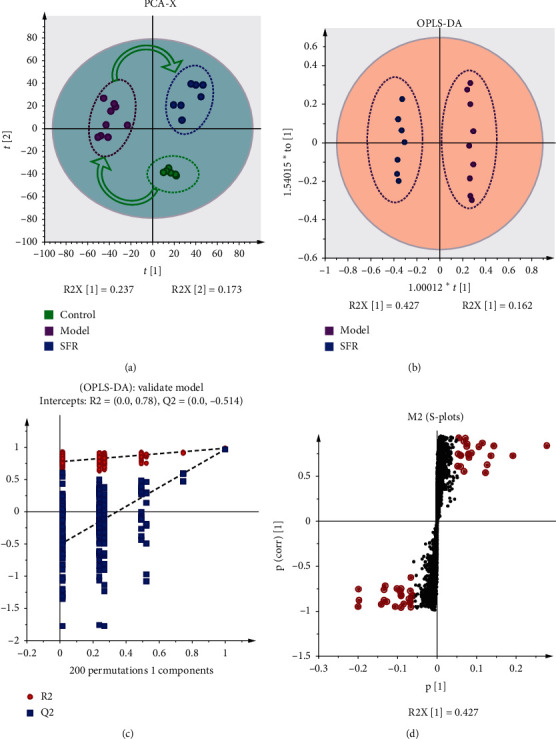
UPLC-HRMS Data analysis. (a) PCA result showing a good separation among control, model, and SFR groups, as well as a metabolic reverse under SFR treatment. (b) OPLS-DA result showing a good separation between model and SFR groups. (c) Robustness assessment of the OPLS-DA model. (d) S-plot analysis for screening differential metabolites.

Nontargeted LC-MS was applied to detect the metabolic changes induced by CKD modeling and SFR treatment. According to the principal component analysis (PCA) result (Figure 2(a)), all samples were located in the 95% confidence interval (Hotelling T2 ellipse) along with a good separation among control, model, and SFR groups (R2X = 0.663). The cumulative values R2Y and Q2 were all greater than 0.6 and the Eigenvalue was 1.36. The result indicated that the model was stable and reliable, and the intervention of SFR can cause significant changes in the related components. Further, in order to eliminate the noise influence which is not related to the study, Orthogonal-PLS Data Analysis (OPLS-DA) was used to screen the variables responsible for the differences among the three groups (Figure 2(b)). With all the samples located in the 95% confidence interval (Hotelling T2 ellipse), and cumulative values R2Y and Q2 being greater than 0.6 (R2X = 0.651 and R2Y = 0.998), it is suggested that OPLS-DA explains the difference between model and SFR groups well and a powerful effect of SFR on CKD. Moreover, the robustness of the OPLS-DA model was assessed by a 200 times permutation test and no overfitting was observed (Figure 2(c)). As illustrated by S-plot (Figure 2(d)), 98 ions, which contribute to the good separation among groups, were selected with VIP > 1 in ESI+, *p* < 0.05 in unidimensional *t*-test for chemical structure identification.

### 3.3. Identification of Metabolites

Compound Discoverer 2.0 software was used for structure mapping operation based on databases including mzCloud, Chemspider, and KeggPathways with accurate MS1 data. Automatic mapping analysis was performed with parameters for the quality as follows: MS1 accuracy <5 PPM, HighChemLow + HighRes applied for spectral library search algorithm, cutoff with mapping score >60, and isotope matching and background noise deducting being applied. Unassigned metabolites were further uploaded to reliable online databases like Metlin (http://metlin.scripps.edu/) and Human Metabolome Database (http://www.hmdb.ca/) for further mapping. The metabolites identified by the above strategy were further identified by manual debris identification and 44 structures were identified (Table 2).

### 3.4. Metabolic Regulation Exploration

**Figure 3:**
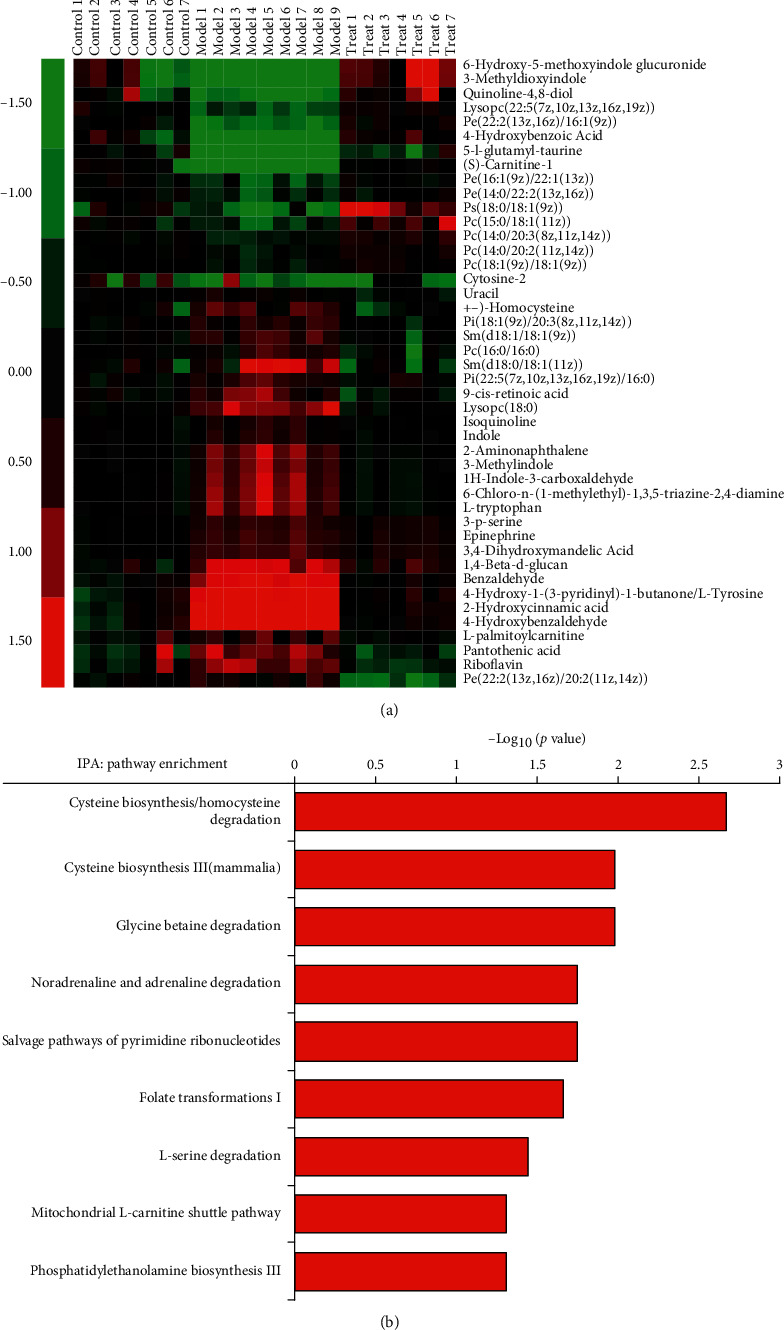
Bioinformatics analysis based on differential metabolites. (a) Heat map showing HCA result. (b) IPA pathway analysis result.

First, hierarchical clustering analysis (HCA), a preliminary bioinformatic analysis, was performed on the differential metabolites, and a heat map was obtained based on the Euclidean distance (Figure 3(a)). Among the differential metabolites, 28 were increased and 17 were decreased responding to CKD modeling. These changes were significantly reversed under drug intervention, indicating the important role of these differential metabolites in biological functions and drug regulation. On the basis of the identified metabolites, biological functions and pathways were enriched with IPA software. It was found that the metabolic regulations of SFR on CKD mainly involve in pathways of glycine biosynthesis I, mitochondrial L-carnitine shuttle pathway, phosphatidylethanolamine biosynthesis III, sphingosine-1-phosphate signaling, L-serine degradation, folate transformations I, noradrenaline and adrenaline degradation, salvage pathways of pyrimidine ribonucleotides, cysteine biosynthesis III (Mammalia), glycine betaine degradation, and cysteine biosynthesis/homocysteine degradation (Figure 3(b)).

**Figure 4:**
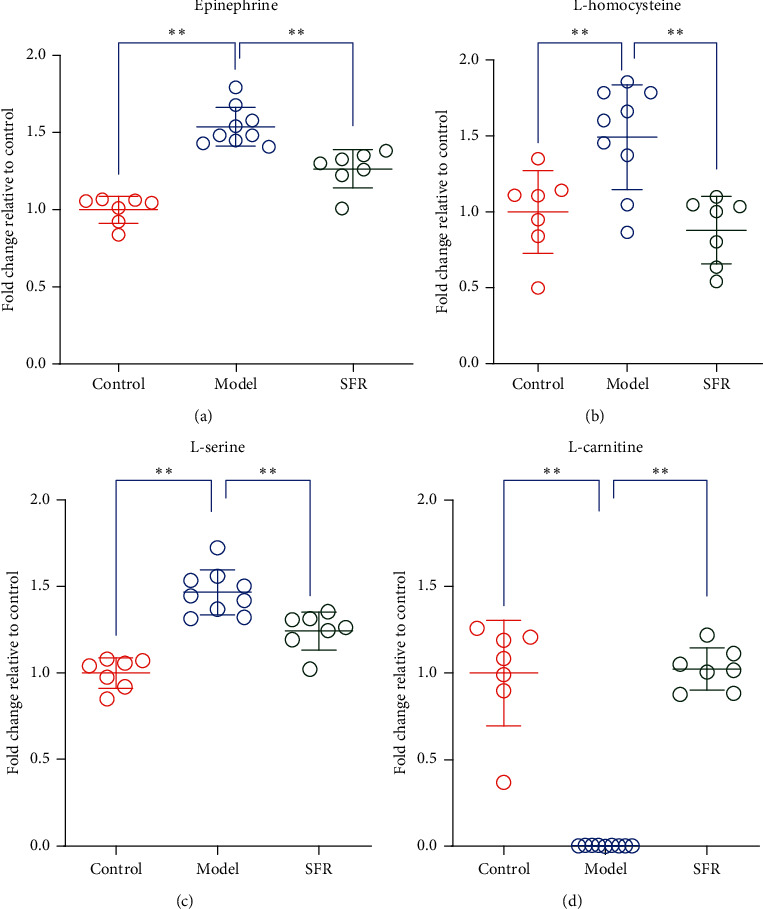
(a–d) Production variations of epinephrine, L-homocysteine, L-serine, and L-carnitine in the serum of rats, which diverted significantly in SFR's regulation. Data are presented as mean ± SD, *t*-test, ^*∗∗*^*p* < 0.01.

**Figure 5:**
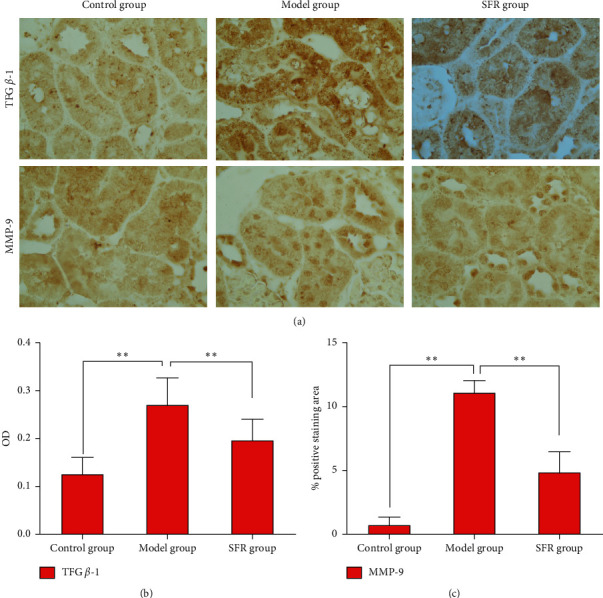
Expression variations of TGF*β*-1 and MMP-9 in kidneys of different-treated rats. (a) Immunohistochemical staining results. (b) OD value for TGF*β*-1 expression evaluation. (c) Positive staining area for MMP-9 expression evaluation. Data are presented as mean ± SD, *t*-test, ^*∗∗*^*p* < 0.01.

By performing network analysis with IPA, TGF*β*-1 and MMP-9 were predicted as links which play key roles in the drug regulation along with altered expressions of 1-oleoyl lysophosphatidylcholine, choline, epinephrine, L-serine (Figure 4(c)), L-homocysteine (Figure 4(b)), L-alpha-lysophosphatidylcholine, stearoyl, and L-tryptophan. Therefore, we give further investigation into the expression variations of TGF*β*-1 and MMP-9 responding to CKD modeling and SFR treatment by applying immunohistochemical staining (Figure 5(a)). The expression of TGF*β*-1 can be determined according to the OD and distribution area. The expression of MMP-9 expression was evaluated based on the percentage of the positively stained area. The result showed that the upregulation of the expression of TGF*β*-1 and MMP-9 in kidney tissues induced by CKD modeling was significantly reversed under SFR treatment (Figures 5(b) and 5(c)). Downregulated MMP-9 indicated a better condition of the kidney. These results suggested the anti-interstitial fibrosis function of SFR by reducing the expression of TGF*β*-1 and interfering with the metabolism of homocysteine.

Moreover, by inspecting the raw data, SFR showed not only dramatical upregulation on L-carnitine (Figure 4(d)) but also reverse on increased epinephrine (Figure 4(a)), which indicate a great help of recovering from CKD by improving renal microvascular status.

## 4. Discussion

CKD is becoming a health problem worldwide which is an economic burden on the society and families with the morbidity and mortality growing every year. CKD ultimately ends in renal failure without timely and effective treatment. Hyperuricemia is common in kidney disease along with decreased uric acid clearance. It was considered not only a marker of kidney damage but also an independent risk factor for kidney disease progression [23]. Uric acid activates not only kinases like p38 and Erk 1/2 but also nuclear transcription factors like NFkB and AP-1 to trigger an inflammatory response in vascular smooth muscle cells [24, 25]. Besides, the increased uric acid content in the blood was observed associated with the upregulation of proinflammatory factors like IL-1*β*, IL-6, and TNF-*α* [26]. Taken together, CKD is a chronic, complex disease with multiple complications including hypertension, fibrosis, and anemia [27–29].

TCM has shown attractive effects in treating chronic, complex diseases. SFR, a modified prescription of traditional medicine in China, was observed to suppress the growth of multiple markers of CKD in clinical practice. With long-term adherence, which means taking the drugs exactly as prescribed, on time, and following dietary restrictions, patients probably keep blood pressure, urinary protein, SUA, and SCr away from growing, even reduced and kept in relatively low levels, accompanied with GFR and hemoglobin maintained at a certain level. In this study, by employing a CKD rat model that mimics urate nephropathy, we have observed the potent effects of SFR in treating chronic kidney injury. Histopathological damage which occurred in kidneys of CKD modeling rats including expanded or atrophied kidney tubules, inflammatory cell infiltration and fibrosis in tubulointerstitium, glomerular sclerosis, and severely dilated capillaries with sinusoid formation was found significantly relieved by SFR administration. The content of SCr and SUA in blood was also significantly downregulated.

The metabolic changes in serum responding to CKD modeling and SFR treatment were detected with UPLC-HRMS. 98 ions which contribute to the good separation among groups were selected for chemical structure identification. By mapping structures based on mzCloud, Chemspider, and KeggPathways database with accurate MS1 data, a total of 44 differential metabolites were identified with Compound Discoverer 2.0 software. Metabolic pathways were enriched in glycine biosynthesis I, mitochondrial L-carnitine shuttle pathway, phosphatidylethanolamine biosynthesis III, sphingosine-1-phosphate signaling, L-serine degradation, folate transformations I, noradrenaline and adrenaline degradation, salvage pathways of pyrimidine ribonucleotides, cysteine biosynthesis III (Mammalia), glycine betaine degradation, and cysteine biosynthesis/homocysteine degradation. These findings strongly suggest that SFR exerts its potent protective effect against glomerular sclerosis and interstitial fibrosis in the kidney by interfering with homocysteine metabolism.

In the methionine cycle, homocysteine is an intermediate product which is derived from methionine, and it is cleaved into cysteine via the transsulfuration pathway. Homocysteine metabolism reported mainly occurs in the kidney and exerts a great impact on renal physiology [30–34]. About 20% of homocysteine failed to bound to proteins in plasma and subjected to glomerular filtration and tubular resorption [35]. Excessive production and accumulation of homocysteine aggravate kidney condition mainly by inducing imbalance of homeostasis and cellular redox and resulting in severe oxidative stress. Along with vasoconstriction and renal microvasculature impairment, homocysteine was further accumulated in turn, thereby resulting in chronic renal disorder [36–39]. Moreover, TGF*β*-1 is a prosclerotic cytokine involved in the extracellular matrix maintenance and synthesis of integrin matrix receptors [40]. Increased TGF*β*-1 was generally observed in human glomerulopathies related cases [41–43]. Overproduction of TGF*β*-1 can result in pathological tissue fibrosis [44]. In mouse aortic endothelial cells, myofibroblast differentiation was observed induced by homocysteine-mediated TGF*β*-1 upregulation [45]. MMP-9 is observed upregulated in various nephropathies because of its promotive effect on the development of fibrin-induced glomerular lesions [46, 47]. In this study, the increased expression of TGF*β*-1 and MMP-9 in kidney tissues induced by CKD modeling was observed significantly downregulated under SFR treatment, suggesting that SFR potently inhibits the progression of chronic kidney injury by regulating homocysteine metabolism and disturbing the expression of TGF*β*-1. By inspecting the raw data, SFR showed a dramatic upregulation of L-carnitine, which is conducive to restoring renal function and maintaining erythrocyte cell membrane stabilization [48, 49] and observed to have a beneficial effect on renal anemia [50]. Epinephrine is considered closely related to pathogenesis hypertension, one of the common complications of CKD [51, 52]. SFR also showed a significant reverse on the increased epinephrine in the model group, which indicates a great help of recovering from CKD.

Nonetheless, this research is subjected to several limitations. First, except for SCr and SUA, it would be better to quantify xanthine oxidase activity, kidney rate transport, blood pressure, and urine protein to evaluate the renal function between groups. Second, there was a lack of negative controls in the staining part, which can cause background noise to be undefined. Third, the conclusion of this study was drawn mainly based on metabolomics data, and the true regulatory mechanism of SFR is still some way off. SFR's regulations on protein and gene levels need further exploration. To sum up, this study provides an explanation for SFR's protective effects on CKD, but it is not the most perfect one and there is much more to do.

## 5. Conclusion

**Figure 6:**
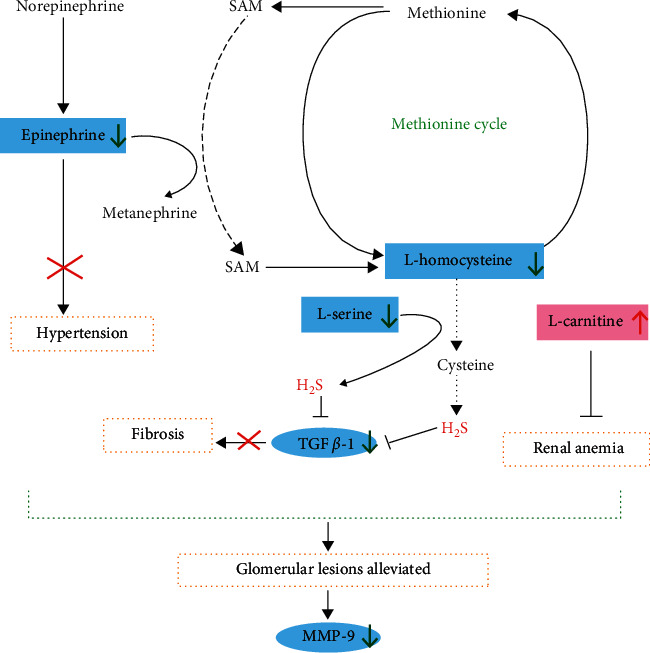
Integrated metabolic regulations of SFR on CKD rats.

In this study, a UPLC-HRMS-based serum metabolomic approach was developed to explore CKD associated metabolic alterations, as well as the intervention mechanism of SFR. Along with 44 differential metabolites identified, SFR was found to exert potent effects of alleviating glomerular sclerosis and interstitial fibrosis in the kidney, mainly via integrated regulations on the metabolism of homocysteine, L-carnitine, and epinephrine, as well as the expression of TGF*β*-1 (Figure 6). This study provides evidence for SFR's protective effects on CKD and reveals the metabolic mechanism behind these benefits.

## Figures and Tables

**Table 1:** The stability data of the UPLC-MS method.

*t*_R_-*m/z*	Precision (RSD, %)		Stability (RSD, %)	
	*t*_R_	Peak intensity	*t*_R_	Peak intensity
2.87-442.52	0.07	1.28	0.25	1.11
5.46-232.63	0.02	1.77	0.10	1.35
9.47-346.26	0.29	1.83	0.15	1.45
12.46-556.29	0.34	3.68	0.27	1.85

**Table 2:** Production changes of differential metabolites which were detected by UPLC-HRMS and screened with Student's *t*-test.

No.	Retention-time	Formula	Name	Ratio (model/control)	Ratio (SFR/model)	*p* value (model/control)	*p* value (SFR/model)
1	3.623	C_7_H_15_NO_3_	L-carnitine	0.003537	225.13066	1.089*E* − 07	3.966*E* − 13
2	10.951	C_4_H_9_NO_2_S	L-homocysteine	1.4923497	0.4590147	0.00791	0.0011462
3	0.683	C_18_H_32_O_18_	1,4-beta-d-glucan	2.2080128	0.4730602	1.508*E* − 06	7.271*E* − 05
4	7.896	C_9_H_7_NO	1H-indole-3-carboxaldehyde	1.8008591	0.3787874	6.835*E* − 05	1.383*E* − 05
5	7.896	C_10_H_9_N	2-aminonaphthalene	1.7888843	0.3995305	2.87*E* − 05	9.924*E* − 06
6	8.019	C_9_H_8_O_3_	2-hydroxycinnamic acid	4.102932	0.2163455	2.432*E* − 07	3.139*E* − 07
7	4.669	C_8_H_8_O_5_	3,4-dihydroxymandelic acid	1.5635809	0.6384346	1.316*E* − 07	0.000609
8	4.907	C_9_H_9_NO_2_	3-methyldioxyindole	0.0617987	28.545473	0.0003882	0.0001272
9	7.896	C_9_H_9_N	3-methylindole	1.726946	0.3973962	5.069*E* − 05	9.508*E* − 06
10	6.06	C_3_H_8_NO_6_P	L-serine	1.4661334	0.6594405	1.218*E* − 06	0.0028474
11	8.01	C_9_H_11_NO_3_	L-tyrosine	3.0930061	0.2851314	1.762*E* − 07	2.003*E* − 07
12	8.019	C_7_H_6_O_2_	4-hydroxybenzaldehyde	4.8105695	0.1788097	2.149*E* − 07	2.108*E* − 07
13	11.157	C_7_H_6_O_3_	4-hydroxybenzoic acid	0.1746762	5.7697183	3.787*E* − 05	2.714*E* − 09
14	8.978	C_7_H_14_N_2_O_6_S	5-l-glutamyl-taurine	0.0276501	20.728663	2.098*E* − 08	3.493*E* − 05
15	7.896	C_6_H_10_ClN_5_	6-chloro-n-(1-methylethyl)-1,3,5-triazine-2,4-diamine	1.8510126	0.3676839	5.818*E* − 05	1.27*E* − 05
16	4.97	C_12_H_7_NO_8_	6-hydroxy-5-methoxyindole glucuronide	0.0286254	62.126321	0.0003199	0.0001008
17	0.734	C_20_H_28_O_2_	9-cis-retinoic acid	1.6116245	0.4233558	0.0019205	0.0005299
18	8.002	C_7_H_6_O	Benzaldehyde	2.5762984	0.3179199	1.163*E* − 07	1.252*E* − 07
19	3.456	C_4_H_5_N_3_O	Cytosine	0.5529155	0.8961198	0.1506915	0.7413868
20	1.644	C_9_H_13_NO_3_	Epinephrine	1.5367492	0.6398639	1.695*E* − 07	0.0007399
21	7.896	C_8_H_7_N	Indole	1.3203628	0.5628054	0.00099	0.0002216
22	7.896	C_9_H_7_N	Isoquinoline	1.2759086	0.5900985	0.0042557	0.0011268
23	8.792	C_23_H_45_NO_4_	L-palmitoylcarnitine	1.4179083	0.5049624	0.0329971	0.0029532
24	7.885	C_11_H_12_N_2_O_2_	L-tryptophan	1.8452391	0.3639046	0.0001183	2.284*E* − 05
25	11.922	C_26_H_54_NO_7_P	Lysopc (18:0)	2.0487088	0.3510377	2.022*E* − 05	9.288*E* − 06
26	10.433	C_30_H_52_NO_7_P	Lysopc (22:5 (7z,10z,13z,16z,19z))	0.5879232	1.5815098	4.966*E* − 05	2.871*E* − 08
27	1.57	C_9_H_17_NO_5_	Pantothenic acid	1.892487	0.3008211	0.0035404	2.502*E* − 07
28	8.578	C_42_H_80_NO_8_P	Pc (14:0/20:2(11z,14z))	0.8523078	1.1325779	0.028192	5.716*E* − 06
29	8.583	C_42_H_78_NO_8_P	Pc(14:0/20:3(8z,11z,14z))	0.7721801	1.346384	0.0023297	4.262*E* − 08
30	8.606	C_41_H_80_NO_8_P	Pc(15:0/18:1(11z))	0.6693928	1.8392962	0.0037784	3.122*E* − 05
31	8.713	C_40_H_80_NO_8_P	Pc(16:0/16:0)	1.3414214	0.4943378	0.0106971	0.0049318
32	8.5	C_44_H_84_NO_8_P	Pc(18:1(9z)/18:1(9z))	0.8360294	1.076322	0.00924	0.0004752
33	8.616	C_41_H_78_NO_8_P	Pe(14:0/22:2(13z,16z))	0.6923289	1.1487602	0.0008253	0.0007498
34	8.54	C_43_H_82_NO_8_P	Pe(16:1(9z)/22:1(13z))	0.6367583	1.1973036	0.0005998	0.0006618
35	8.547	C_43_H_80_NO_8_P	Pe(22:2(13z,16z)/16:1(9z))	0.4371836	1.9951094	8.633*E* − 07	7.816*E* − 07
36	8.333	C_47_H_86_NO_8_P	Pe(22:2(13z,16z)/20:2(11z,14z))	1.2159935	0.3261839	0.1020484	3.323*E* − 05
37	5.649	C_47_H_83_O_13_P	Pi(18:1(9z)/20:3(8z,11z,14z))	1.3752143	0.5520982	0.0010777	0.0007957
38	5.672	C_47_H_81_O_13_P	Pi(22:5(7z,10z,13z,16z,19z)/16:0)	1.4501963	0.5528191	0.0006161	0.0022218
39	8.929	C_42_H_80_NO_10_P	Ps(18:0/18:1(9z))	0.5130888	3.3917303	0.0111159	4.035*E* − 05
40	5.737	C_9_H_7_NO_2_	Quinoline-4,8-diol	0.3408319	3.9021513	0.0038625	0.0017152
41	2.606	C_17_H_20_N_4_O_6_	Riboflavin	1.8788114	0.2936902	0.0006979	4.67*E* − 08
42	9.62	C_41_H_83_N_2_O_6_P	Sm(d18:0/18:1(11z))	2.0206224	0.3173536	0.00779	0.0029009
43	9.632	C_41_H_81_N_2_O_6_P	Sm(d18:1/18:1(9z))	1.5075415	0.4668141	0.0001188	4.025*E* − 05
44	7.343	C_4_H_4_N_2_O_2_	Uracil	1.117015	0.5900336	0.1148186	0.0024045

## Data Availability

All data generated or analyzed during this study are included in this article.
